# Species of *Annulotrema* (Monopisthocotylea, Dactylogyridae) parasitising African tetras (Characiformes, Alestidae) in the Phongolo River, South Africa with the description of four new species[Fn FN1]

**DOI:** 10.1051/parasite/2024066

**Published:** 2024-10-31

**Authors:** Maria Lujza Kičinjaová, Iva Přikrylová, Mária Seifertová, Eva Řehulková, Milan Gelnar, Nico J. Smit

**Affiliations:** 1 Department of Botany and Zoology, Faculty of Science, Masaryk University Kotlářská 2 61137 Brno Czech Republic; 2 Water Research Group, Unit for Environmental Sciences and Management, North-West University Private Bag X6001 Potchefstroom 2520 South Africa; 3 DSI-NRF SARChI Chair (Ecosystem Health), Department of Biodiversity, School of Molecular and Life Sciences, University of Limpopo Private Bag X1106 Sovenga 0727 South Africa

**Keywords:** Monogenea, 28S rDNA, South Africa, Alestidae, *Annulotrema*

## Abstract

Species of Alestidae are known to be parasitised by dactylogyrid monogeneans representing three genera, *Afrocleidodiscus* Paperna, 1969, *Annulotrema* Paperna & Thurston, 1969, and *Characidotrema* Paperna & Thurston, 1968. The objective of the present study was to investigate the species diversity of Monopisthocotylea of African tetras from the Lower Phongolo River and floodplain in South Africa. Four new and two previously described species of *Annulotrema* were identified from the gills of three species of African tetras, *Brycinus imberi*, *Hydrocynus vittatus*, and *Micralestes acutidens*. The collected parasites were studied using two complementary approaches: morphology of hard sclerotised structures, and molecular markers using rDNA sequence data (28S rDNA, 18S rDNA, and ITS1). Three new species, *Annulotrema arcum* n. sp., *Annulotrema caputfemoris* n. sp., and *Annulotrema strepsiceros* n. sp., were described from *B. imberi* and one species, *Annulotrema retortum* n. sp., from *M. acuditens*. Two previously described species, *Annulotrema pikoides* Guégan, Lambert and Birgi, 1988 and *A. pseudonili* Kičinjaová and Řehulková, 2017, were newly recorded from *H. vittatus* in South Africa. *Annulotrema arcum* n. sp. and *A. caputfemoris* n. sp. share similar male copulatory organ morphologies, suggesting a close phylogenetic relationship as sister taxa. Despite weak nodal support, *A. strepsiceros* n. sp. shows morphological congruence with the former two species, reinforcing their molecular linkage. The present study shows a critical need for the exploration of monopisthocotylean diversity and the paucity of available molecular data of representatives from this group.

## Abstract (fr)

Les espèces d'Alestidae sont connues pour être parasitées par des monogènes Dactylogyridae représentant trois genres, *Afrocleidodiscus* Paperna, 1969, *Annulotrema* Paperna & Thurston, 1969 et *Characidotrema* Paperna & Thurston, 1968. L'objectif de la présente étude était d'examiner la diversité des espèces de Monopisthocotylea des tétras africains du cours inférieur de la rivière Phongolo et de sa plaine inondable en Afrique du Sud. Quatre nouvelles espèces d'*Annulotrema* et deux espèces déjà décrites ont été identifiées à partir des branchies de trois espèces de tétras africains, *Brycinus imberi*, *Hydrocynus vittatus* et *Micralestes acutidens*. Les parasites collectés ont été étudiés en utilisant deux approches complémentaires, la morphologie des structures sclérifiées et les marqueurs moléculaires utilisant des données de séquence d'ADNr (ADNr 28S, ADNr 18S et ITS1). Trois nouvelles espèces, *Annulotrema arcum* n. sp., *Annulotrema caputfemoris* n. sp. et *Annulotrema strepsiceros* n. sp., ont été décrites chez *B. imberi* et une espèce, *Annulotrema retortum* n. sp., chez *M. acuditens*. Deux espèces précédemment décrites, *Annulotrema pikoides* Guégan, Lambert et Birgi, 1988 et *A. pseudonili* Kičinjaová et Řehulková, 2017, ont été nouvellement répertoriées chez *H. vittatus* en Afrique du Sud. *Annulotrema arcum* n. sp. et *A. caputfemoris* n. sp. partagent des morphologies similaires de l’organe copulateur mâle, suggérant une relation phylogénétique étroite de taxons frères. Malgré un faible support nodal, *A. strepsiceros* n. sp. présente une congruence morphologique avec les deux premières espèces, renforçant leur lien moléculaire. La présente étude montre qu'il est essentiel d'explorer la diversité des Monopisthocotylea et que les données moléculaires disponibles sur les représentants de ce groupe sont rares.

## Introduction

African tetras (Characiformes: Alestidae) represent the most speciose group of African characiform fishes with 120 currently known species belonging to 21 genera [13]. To date, only 16 species of African tetras represented by genera *Alestes* Müller and Troschel*, Brycinus* Valenciennes*, Hemigrammopetersius* Pellegrin*, Hydrocynus* Cuvier*, Micralestes* Boulenger and *Phenacogrammus* Eigenmann, are known to be parasitised by dactylogyrid monopisthocotyleans. These species of parasites (57 spp.) are classified into three genera, i.e. *Afrocleidodiscus* Paperna, 1969 (1 species), *Annulotrema* Paperna and Thurston, 1969 (43 spp.), and *Characidotrema* Paperna and Thurston, 1968 (13 spp.) [23, 24, 25, 47, 48].

Moreover, six other species of three genera representing two families have been reported from African alestid hosts. These are one species of *Diplozoon* von Nordmann, 1832 (Diplozoidae Palombi, 1949) [36, 57], four species of *Afrogyrodactylus* Paperna, 1968, and one species of *Gyrodactylus v*on Nordmann, 1833, both genera belonging to Gyrodactylidae van Beneden and Hesse, 1832 [35, 42, 43].

Freshwater bodies of Southern Africa harbour six species of African tetras, *Brycinus imberi* (Peters), *Brycinus lateralis* (Boulenger), *Hemigrammopetersius barnardi* (Herre), *Hydrocynus vittatus* Castelnau, *Micralestes acutidens* (Peters), and *Rhabdalestes maunensis* (Fowler) [53]. The tigerfish, *H. vittatus* is one of the most studied species of the South African ichthyofauna. It is important for both commercial and recreational fisheries [16, 51, 54, 60], and it is a good model fish to be used as a bio-indicator [9, 14, 30, 59]. In 2007, tigerfish was included on South Africa’s protected and threatened species list [8]; therefore, many published studies are related to its conservation and understanding of all biological and ecological aspects of this species [1]. Nevertheless, to date, very few studies have dealt with the monopisthocotylean parasites of Alestidae from South Africa. *Annulotrema pikei* (Price, Peebles and Bamforth, 1969) Paperna, 1979 represent the only species found from the gills of *H. vittatus* (Phongolo flood plain, Natal [41]) and *Characidotrema auritum* Kičinjaová and Řehulková, 2019 is known from *B. imberi* in the Phongolo River, Ndumo [47]. Regarding gyrodactylid parasites from South Africa, the only parasite known so far is *Afrogyrodactylus kingi* Přikrylová and Luus-Powell, 2014 [42], which was found on the gill arches of sharptooth tetra, *M. acutidens*. Nine species/subspecies belonging to *Annulotrema* have been recorded from *H. vittatus* in Mali, Tanzania, and Zimbabwe [17, 24, 37, 38] and the spot-tail, *B. imberi* is known as a host of dactylogyrids from Cameroon, the Democratic Republic of Congo, Tanzania, and Zimbabwe [27, 36, 47] (for full details see Table 1).

**Table 1:** Records of monopisthocotylean parasites from the gills of *Brycinus imberi*, *Hydrocynus vittatus*, and *Micralestes acutidens* (Alestidae).

Host species	Monogenea	Country	Reference
*B. imberi*	*Annulotrema alestesimberi* Paperna, 1973	Tanzania	[36]
	*A. allogravis* Paperna, 1973	Tanzania	[36]
	*Characidotrema auritum* Kičinjaová and Řehulková, 2019	South Africa	[47]
		Zimbabwe	
	*C. ruahae* (Paperna, 1979) Kritsky, Kulo and Boeger, 1987	Tanzania	[27]
	*C. vespertilio* Kičinjaová and Řehulková, 2019	Cameroon	[47]
		DR Congo	
*H. vittatus*	*A. bracteatum* Kičinjaová and Řehulková, 2018	Zimbabwe	[24]
	*A. longipenis* Paperna, 1969	Mali	[17]
	*A. magnum* Paperna, 1973	Tanzania	[37]
	*A. nili* Paperna, 1973	Mali	[17]
	*A. nili ruahae* Paperna, 1979	Mali	[17]
		Tanzania	[38]
	*A. pikei* (Price, Peebles and Bamforth, 1969) Paperna, 1979	South Africa	[41]
	*A. pikei ruahae* Paperna, 1979	Tanzania	[38]
	*A. pikoides* Guégan, Lambert and Birgi, 1988	Mali	[17]
		Zimbabwe	[24]
	*A. pseudonili* Kičinjaová and Řehulková, 2017	Zimbabwe	[24]
	*A. ruahae* Paperna, 1973	Tanzania	[37]
*M. acutidens*	*Afrogyrodactylus kingi* Přikrylová and Luus-Powell, 2014	South Africa	[42]

The present investigation into the monopisthocotylean fauna of African tetras (*B. imberi, H. vittatus*, and *M. acutidens*) from South African freshwaters revealed the presence of two previously described and four new species of *Annulotrema*. Detailed morphological description of these species was complemented by molecular analyses of three ribosomal DNA markers (partial 18S rDNA, internal transcribed spacer 1 (ITS1), and 28S rDNA).

## Materials and methods

### Ethics

Ethical clearance for this work was received from the North-West University AnimCare Research Ethics Committee (NWU-00264-16-A5). Fish handling from the point of collection until dissection was initiated following the NWU-approved protocol (SOP NWU-00272-17-A5) for the temporary holding of ﬁsh. Each ﬁsh was humanely killed by percussive stunning and cervical transection and dissected following the protocol for the Ethical Handling of Ectothermic Vertebrates (SOP NWU-00267-17-A5).

### Fish collection

Alestid hosts were collected at two sites in the Lower Phongolo River and floodplain, South Africa: Broken bridge (Ndumo Game Reserve, the outflow of Nyamithi Pan, 26°52.961833′S, 32°18.678′E), and bridge out (bridge outside of the Ndumo Game Reserve, 27°2.23482′S, 32°16′E) during a field campaign in February 2017. Permits for sampling were obtained from Ezemvelo KZN Wildlife (OP898/2016, OP 899/2016, and OP 1075/2017).

### Parasites preparation

Monopisthocotyleans found on the gills of dissected fishes were collected with fine needles, put in a drop of water on a slide, and covered by a cover slip. Specimens prepared for the morphological study were flattened using coverslip pressure and fixed with a mixture of glycerine and ammonium picrate (GAP [29]). Specimens used for the molecular study were bisected; the anterior body part with the reproductive system was mounted into GAP or Hoyer’s medium [26] for species determination, and the posterior body part was individually preserved in 96% ethanol for DNA extraction.

### Taxonomical evaluation

The taxonomical evaluation was performed with the aid of a Nikon Eclipse 80i microscope (Nikon, Tokyo, Japan) equipped with phase contrast optics. Measurements, all in micrometres, were taken using microscope imaging software (NIS-Elements, Nikon, Tokyo, Japan); measurements are presented as mean, followed by the range and the number (*n*) of specimens measured in parentheses. The schemes of measurement for the hard structures, i.e. haptoral sclerites, male copulatory organ (MCO), and vagina were used according to Figure 1 in Kičinjaová *et al.* 2017 [24]. Drawings of species new to science were made using an Olympus BX 61 microscope (Olympus, Tokyo, Japan) equipped with phase contrast optics and drawing attachment. The numbering of hook pairs (in Roman numerals) follows that recommended by Mizelle [32]. For comparative purposes, the following specimens of previously described species were examined: *A. alestesnursi* syntypes (M.T. 35.918) from *Alestes nurse* (= *Brycinus nurse*) (Uganda) and *A. allogravis* syntypes (M.T. 35.716) from *Alestes imberi* (= *B. imberi*) (Tanzania) from the Royal Museum for Central Africa, Tervuren, Belgium and *A. pikoides* holotype, paratypes (MNHN 192 HC) from *H. vittatus* (Mali) from the National Museum of Natural History, Paris, France. Note that the new taxa’s authors differ from the authors of this paper; following Article 50.1 and Recommendation 50A of the International Code of Zoological Nomenclature [20]. Type and voucher specimens, and also molecular vouchers (=hologenophores, paragenophores [40]) were deposited in the parasitological collection of the National Museum, Bloemfontein, South Africa (NMBP) and in the Helminthological Collection of the Institute of Parasitology, Biology Centre of the Academy of Sciences of the Czech Republic, in České Budějovice (IPBCAS), remounted prior to deposition into Canada balsam [9]. Epidemiological indices such as prevalence (P), intensity of infection (IF), mean intensity of infection (MI), and mean abundance (MA) were calculated following Bush *et al.* [6].

**Figure 1:**
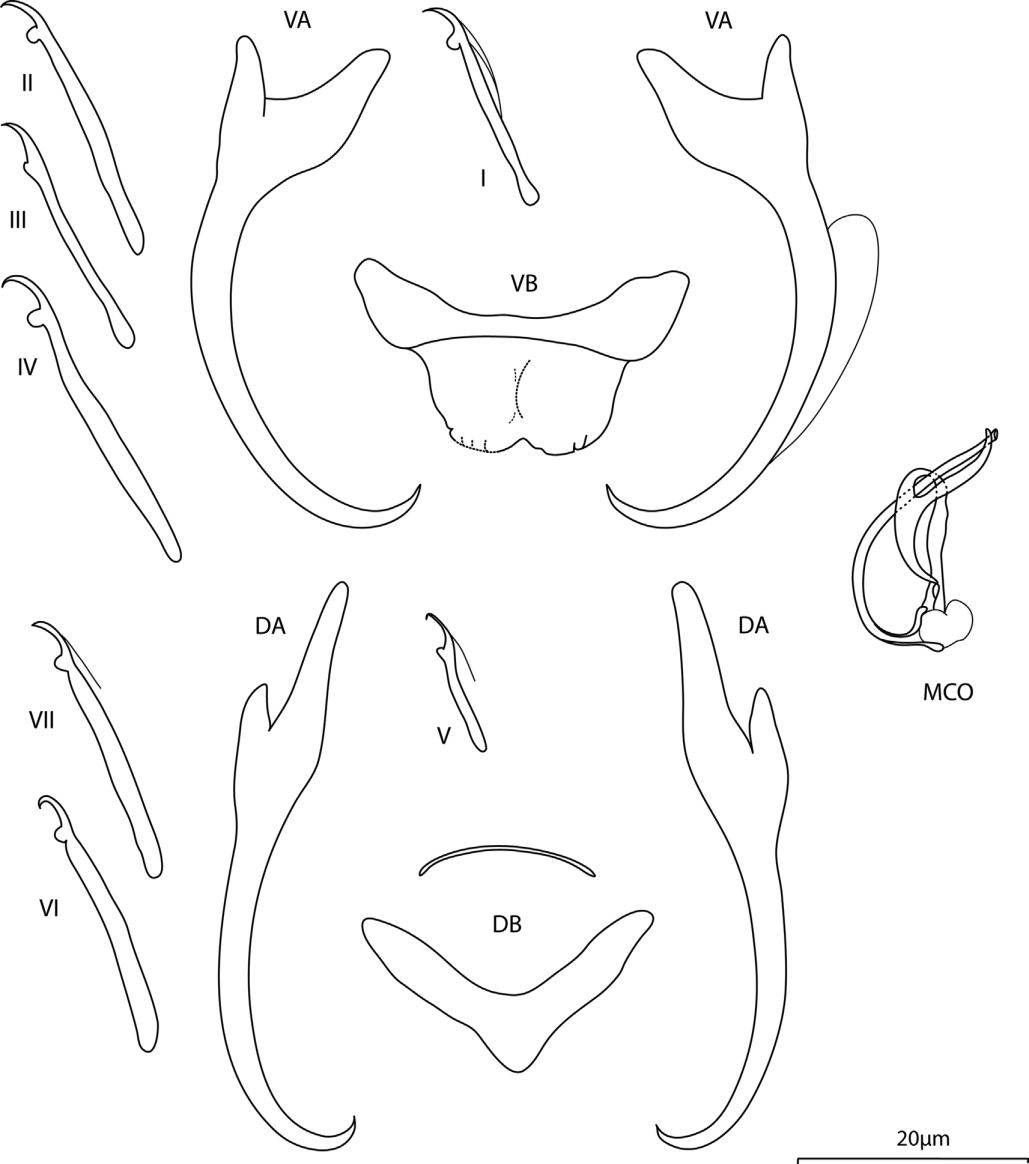
Line drawings of hard parts of *Annulotrema arcum* n. sp. ex *Brycinus imberi.* VA – ventral anchor, VB – ventral bar, DA – dorsal anchor, DB – dorsal bar, MCO – male copulatory organ.

### DNA extraction, amplification, and sequencing

Total genomic DNA was separately extracted from each ethanol-fixed specimen (2–4 specimens per species) using a DNeasy^®^ Blood and Tissue Kit (QIAGEN, Hilden, Germany), following the manufacturerʼs instructions. Two fragments of nuclear ribosomal DNA were analysed: a fragment spanning partial 18S rDNA and internal transcribed spacer 1 (18S-ITS1); and a fragment of partial 28S rDNA (28S). Primer details and thermal cycle profiles are provided in Table 2. Both PCRs were carried out in a total volume of 30 μL containing 5 μL of DNA extract, 1× PCR buffer (Fermentas by Thermo Fisher Scientific, Waltham, MA, USA), 1.5 mM MgCl_2_, 200 µM of each dNTP, 0.5 μM (for 28S) or 0.8 μM (for 18S-ITS1) of each primer, and 1U of Taq polymerase (Fermentas). PCR amplicons were purified using an ExoSAP-IT™ (Affymetrix Inc., Santa Clara, CA, USA) and sequenced directly from both strands using the PCR primers. DNA sequencing was carried out using a BigDye^®^ Terminator v3.1 Cycle Sequencing Kit (Applied Biosystems by Thermo Fisher Scientific, Prague, Czech Republic) and an Applied Biosystems 3130 Genetic Analyzer (Applied Biosystems). Sequences were assembled and edited using Sequencher software (Gene Codes Corp., Ann Arbor, MI, USA). Basic Local Alignment Search Tool (BLAST; https://blast.ncbi.nlm.nih.gov/Blast.cgi) searches were performed to verify the sequence similarity to species of dactylogyrid genera.

**Table 2:** List of primers used for PCR amplification of nuclear markers in the present study.

rDNA fragment	Primer name	Direction	Sequence (5′–3′)	PCR thermal profile	Product size	Reference
18S + ITS1	S1	Forward	ATT CCG ATA ACG AAC GAG ACT	94 °C:2 min; 39 × (94 °C: 60 s, 53 °C: 60 s, 72 °C: 90 s); 72 °C: 10 min	~ 1000 bp	[52]
	IR8	Reverse	GCT AGC TGC GTT CTT CAT CGA			[55]
28S	C1	Forward	ACC CGC TGA ATT TAA GCA	94 °C:2 min; 39 × (94 °C: 20 s, 56 °C: 30 s, 72 °C: 90 s); 72 °C: 10 min	~ 800 bp	[19]
	D2	Reverse	TGG TCC GTG TTT CAA GAC			[19]

### Molecular data analysis

Alignments of newly generated sequences of 18S, ITS1, and 28S rDNA fragments were generated using MAFFT [22] and manually adjusted in BioEdit [18]. Interspecific genetic distances were determined using distance matrices (uncorrected *p*-distances) in MEGA 11 [56] separately for each fragment. Phylogenetic analyses were performed on the 28S rDNA dataset using maximum likelihood (ML) and Bayesian inference (BI) methods. ML analysis was conducted using the IQ-TREE [34] on the W-IQ-TREE web server [58]. Based on the preliminary results investigating a broader phylogeny of dactylogyrid genera of alestid hosts (unpublished), *Characidotrema nursei* Ergens, 1973 (accession number MK012540) a parasite of the Nurse tetra, *Brycinus nurse* (Rüppell) in Sudan, was used as the outgroup. ModelFinder [21] was used to identify the best substitution model (TIM3+F+G4). Branch support was estimated using ultrafast bootstrap approximation [31] with 1000 replicates. BI analysis was performed in MrBayes 3.2.1 [45] and was run for 1 million generations, sampling every 100 generations, with a burn-in of 25%. Generated trees were visualised and edited in FigTree ver. 1.4.3 [44].

## Results

Three species of African tetras, i.e. *B. imberi* (*n* = 10), *H. vittatus* (*n* = 6), and *M. acutidens* (*n* = 20), were collected and dactylogyrid monopisthocotylean were found on the gills. Taxonomic evaluation of the monopisthocotylean isolated from the gills revealed the presence of six *Annulotrema* spp*.* Three species were identified from *B. imberi*; two species from *H. vittatus*, and one species from *M. acutidens*. The *Annulotrema* spp. from *B. imberi* and *M. acutidens* represent taxa new to science. Full morphometric descriptions are presented for four newly identified species. For the two known species from *H. vittatus*, *A. pikoides*, and *A. pseudonili*, line drawings are not included as they were recently presented by Kičinjaová *et al.* 2017 [24]. The collection sites in the Lower Phongolo River and floodplain in South Africa represent new locality records for all known and new species described herein.

Dactylogyridae Bychowsky, 1933

*Annulotrema* Paperna and Thurston, 1969

## *Annulotrema pikoides* Guégan, Lambert and Birgi, 1988

*Type host: Hydrocynus vittatus* (Castelnau, 1861)

*Type locality:* Mali

*Other records: H. vittatus*, Lake Kariba, Zimbabwe (16°4′51.63″S; 28°52′4.98″E) [24]

*Present record: H. vittatus,* Phongolo River, KwaZulu-Natal Province, South Africa: Broken bridge (Ndumo Game Reserve, outflow of Nyamithi Pan, 26°52.961833′S, 32°18.678′E)

*Site:* Gill lamellae

*Type-specimens examined*: Holotype, paratypes (MNHN 192 HC) of *A. pikoides* from *H. vittatus* (Mali).

*Material deposited:*Two voucher specimens, and one paragenophore (IPBCAS M-798), four voucher specimens, and a hologenophore (NMB P-1049, 1051, 1052).

*Representative DNA sequences*: GenBank: PQ279565 (18S-ITS1 rDNA) and PQ279570 (28S rDNA).

*Infestation indices*: *P* = 100%, IF = 1–6, MI = 2.5, MA = 2.5

*Measurements*: Body length 658 (566–746; *n* = 5); greatest width 107 (93–120; *n* = 5). Ventral anchors: inner length 45 (44–46; *n* = 5); outer length 41 (40–43; *n* = 5); inner root 12 (9–13; *n* = 5); outer root 5 (4–7; *n* = 5); point 7 (6–8; *n* = 5). Dorsal anchors: inner length 46 (44–50; *n* = 5); outer length 39 (37–43; *n* = 5); inner root 15 (14–15; *n* = 5); outer root 6 (5–6; *n* = 5); point 6 (6–8; *n* = 5). Ventral bar: total length 31 (30–32; *n* = 5); width 6 (4–8; *n* = 5); total width 13 (11–17; *n* = 5). Dorsal bar: total length 28 (27–29; *n* = 5); width 8 (8–9; *n* = 5); total width 12 (11–13; *n* = 5). Hooks 7 pairs, dissimilar in size; hook lengths (*n* = 4): pair I 16 (13–18); pair II 22 (21–22); pair III 27 (25–30); pair IV 33 (32–33); pair V 12 (9–14); pair VI 25 (22–27); VII 22 (21–24). MCO: total length 57 (53–64; *n* = 5); tube-trace length 72 (68–75; *n* = 5); base length 7 (7–8; *n* = 5); base width 4 (3–5; *n* = 5). Vagina not observed.

*Molecular characterisation:* Fragments of 18S-ITS1 (792 bp), and 28S rDNA (758 bp) regions were successfully sequenced for three specimens of *A. pikoides*. The 18S-ITS1 and 28S rDNA sequences obtained for this species did not show any intraspecific variation.

### Remarks

The present specimens of *A. pikoides* correspond well in morphological characters to those in the original description of *A. pikoides* by Guégan *et al.* [17]. Comparison of metrical characters of our specimens, type specimens, and specimens found by Kičinjaová *et al.* [24] in Zimbabwe revealed minor differences in the length of the copulatory tube (68–75, 74–101, and 95–105 respectively). The vagina is not visible in the present specimens or type material. In specimens of *A. pikoides* described in Kičinjaová *et al.* [24], the vagina was observed but poorly sclerotised. Unfortunately, no molecular data from Kičinjaová *et al.* [24] are available to reject or confirm the intraspecific variability in *A. pikoides* within specimens from South Africa and Zimbabwe. Nevertheless, the shape of the MCO resembling open handcuffs is an unmistakable characteristic confirming the co-specificity of all the mentioned species.

## *Annulotrema pseudonili* Kičinjaová and Řehulková, 2017

urn:lsid:zoobank.org:act:AEA4DC34-2367-4C84-B5C2-C28E1FFF3780

*Type host: Hydrocynus vittatus* (Castelnau, 1861)

*Type locality:* Lake Kariba, Zimbabwe (16°4′51.63″S; 28°52′4.98″E) [24]

*Present record: H. vittatus*, Phongolo River, South Africa: Broken bridge (Ndumo Game Reserve, outflow of Nyamithi Pan, 26°52.961833′S, 32°18.678′E)

*Site:* Gill lamellae

*Material deposited:* Four voucher specimens, one paragenophore (IPBCAS M-799), eight voucher specimens, and hologenophore (NMB P-1048, 1049, 1050).

*Representative DNA sequences*: GenBank: PQ279566 (18S-ITS1 rDNA) and PQ279571 (28S rDNA).

*Infestation indices*: *P* = 100%, IF = 2–11, MI = 4, MA = 4

*Measurements*: Body length 593 (480–706; *n* = 5); greatest width 167 (135–187; *n* = 5). Ventral anchors: inner length 40 (39–42; *n* = 6); outer length 46 (43–50; *n* = 6); inner root 12 (11–13; *n* = 6); outer root 6 (4–8; *n* = 6); point 9 (8–10; *n* = 6). Dorsal anchors: inner length 50 (48–53; *n* = 6); outer length 40 (37–42; *n* = 5); inner root 21 (19–23; *n* = 5); outer root 6 (5–8; *n* = 5); point 8 (7–10; *n* = 5). Ventral bar: total length 32 (27–34; *n* = 6); width 6 (4–9; *n* = 4); total width 20 (18–23; *n* = 5). Dorsal bar: total length 32 (29–36; *n* = 6); width 14 (14–14; *n* = 5); total width 21 (18–23; *n* = 5). Hooks 7 pairs, dissimilar in size; hook lengths (*n* = 4): pair I 18 (17–20); pair II 23 (19–25); pair III 27 (25–28); pair IV 32 (30–34); pair V 15 (13–16); pair VI 27 (26–28); VII 33 (31–35). MCO: total length 31 (24–39; *n* = 5); tube-trace length 82 (73–92; *n* = 6); base length 12 (11–13; *n* = 4); base width 5 (5–5; *n* = 3). Vagina not observed.

*Molecular characterisation:* Fragments of 18S-ITS1 (965 bp), and 28S rDNA (755 bp) regions were successfully sequenced for four specimens of *A. pseudonili*. The 18S-ITS1 and 28S rDNA sequences obtained for this species did not show any intraspecific variation.

### Remarks

The present specimens of *A. pseudonili* correspond well with the original description of Kičinjaová *et al.* [24] from the same host in Zimbabwe. South Africa represents a new locality record for this species. Based on the morphology of the MCO and also, haptoral sclerites, *A. pseudonili*, *A. nili*, and *A. nili ruahae* share the same morphotype. In future research, in the light of molecular analysis, their close phylogenetic relationships could be confirmed. The subspecies *A. nili ruahae* should possibly be transferred to the specific rank; however, Kičinjaová *et al.* could not synonymise this species with *A. pseudonili* due to the poor quality of syntypes deposited by Paperna (1979) [23].

## *Annulotrema arcum* Kičinjaová, Přikrylová and Smit n. sp. (Fig. 1)

urn:lsid:zoobank.org:act:A730985F-E47E-46B4-BFE6-225A3D55A9BC

*Type host: Brycinus imberi* (Peters, 1852)

*Type locality:* Phongolo River, South Africa: Broken bridge (Ndumo Game Reserve, outflow of Nyamithi Pan, 26°52.961833′S, 32°18.678′E)

*Site:* Gill lamellae

*Type specimens:* Holotype and two paratype specimens (IPBCAS M-800), three paratype specimens, and hologenophore (NMB P-1061, 1062).

*Representative DNA sequences*: GenBank: PQ279572 (28S rDNA).

*Etymology:* The specific name derived from Latin refers to the arc-shaped MCO, which even with the accessory piece resembles a shooting bow.

*Infestation indices*: *P* = 70%, IF = 2–12, MI = 3.8, MA = 2.7

*Description*: Body length 315 (285–342; *n* = 5); greatest width 131 (95–182; *n* = 5). Ventral anchors with short inner root recurved at its terminal part, well developed outer root, elongated curved shaft, and short point: inner length 49 (44–52; *n* = 5); outer length 48 (42–51; *n* = 5); inner root 12 (11–13; *n* = 5); outer root 5 (4–6; *n* = 5); point 5 (5–6; *n* = 5). Dorsal anchors with elongated inner root having slightly recurved terminal half, well developed outer root, moderately curved shaft, and short point: inner length 54 (45–60; *n* = 5); outer length 41 (36–45; *n* = 5); inner root 17 (14–19; *n* = 5); outer root 4 (3–5; *n* = 5); point 4 (3–5; *n* = 5). Ventral bar yoke-shaped, supporting membrane well defined: total length 26 (23–28; *n* = 5); median width 8 (4–11; *n* = 5); total width 13 (13–14; *n* = 5). Dorsal bar V-shaped, supporting membrane weakly sclerotised: total length 26 (24–27; *n* = 5); median width 6 (5–9; *n* = 5); total width 11 (10–13; *n* = 5). Hooks 7 pairs, dissimilar in size; hook lengths (*n* = 4): pair I 20 (18–21); pair II 30 (29–31); pair III 29 (27–30); pair IV 36 (33–39); pair V 14 (12–16); pair VI 28 (26–30); pair VII 30 (29–33). Vagina not observed. MCO comprising basally articulated copulatory tube, accessory piece; total length 20 (19–21; *n* = 5). Copulatory tube arcuate, pouch-like base with a tear-like basal process; tube-trace length 30 (29–30; *n* = 5); base length 6 (5–7; *n* = 5); base width 4 (4–4; *n* = 5). Accessory piece well sclerotised, with small proximal loop, distally looping the copulatory tube, supporting tip of the accessory piece guiding the distal part of the copulatory tube.

*Molecular characterisation:* For two specimens of *A. arcum* parasitising *B. imberi* only the 28S rDNA fragment (734 bp) was successfully sequenced. The 28S rDNA sequences obtained for this species did not show any intraspecific variation.

### Remarks

*Annulotrema arcum* n. sp. is in its morphology of sclerotised structures close to *A. caputfemoris* n. sp.,; both species possess a very similar MCO with a solid arcuate copulatory tube and relatively rigid basally articulated accessory piece with two loops. However, the subtle morphological differences are evident: thick-walled base in *A. caputfemoris* n. sp. *vs.* pouch-like in *A. arcum* n. sp., shorter copulatory tube in *A. caputfemoris* n. sp. and thus the smaller arc (20 *vs.* 30 in *A. arcum* n. sp.), and distal part of the accessory piece a supporting tip in *A. arcum* n. sp. *vs.* distal part a sheath in *A. caputfemoris* n. sp. Spikes of a distal part of the accessory piece in *A. arcum* n. sp. are visible even at low magnification (using a microscope equipped with phase contrast) as a very dark three-armed bifurcation.

## *Annulotrema caputfemoris* Kičinjaová, Přikrylová and Smit n. sp. (Fig. 2)

urn:lsid:zoobank.org:act:087A056B-D013-4ACB-9EE4-F3D2C9C6B2CD

**Figure 2:**
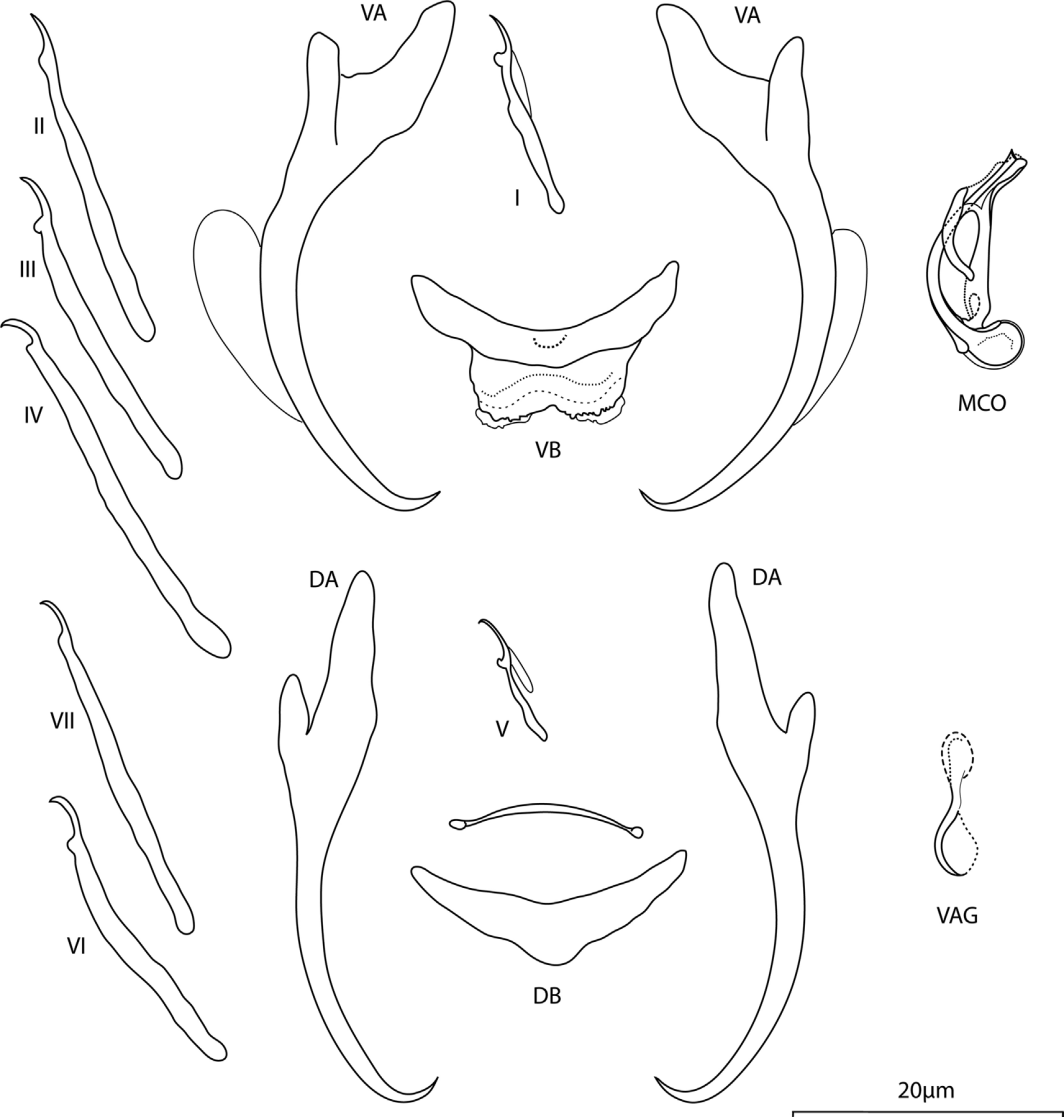
Line drawings of hard parts of *Annulotrema caputfemoris* n. sp. ex *Brycinus imberi.* VA – ventral anchor, VB – ventral bar, DA – dorsal anchor, DB – dorsal bar, MCO – male copulatory organ, VAG – vagina.

*Type host: Brycinus imberi* (Peters, 1852)

*Type locality:* Phongolo River, South Africa: Broken bridge (Ndumo Game Reserve, outflow of Nyamithi Pan, 26°52′57.71″S, 32°18′40.68″E)

*Site:* Gill lamellae

*Type specimens:* Holotype, and four paratype specimens (NMB P-1063, 1064), two paratype specimens, and hologenophore (IPBCAS M-801).

*Representative DNA sequences*: GenBank: PQ279567 (18S-ITS1 rDNA) and PQ279573 (28S rDNA).

*Etymology:* The specific name derived from Latin refers to shape of the MCO resembling the head of the femur – *caput femoris*.

*Infestation indices*: *P* = 70%, IF = 2–7, MI = 2.8, MA = 2

*Description* : Body length 331 (326–340; *n* = 3); greatest width 123 (75–165; *n* = 3). Ventral anchors with short inner root recurved along its terminal part, well developed outer root, elongated curved shaft, and short point, anchor filaments visible: inner length 41 (40–44; *n* = 4); outer length 40 (39–42; *n* = 3); inner root 12 (10–12; *n* = 3); outer root 4 (3–4; *n* = 3); point 4 (4–5; *n* = 3). Dorsal anchors with elongated inner root having recurved terminal half, well developed outer root, moderately curved shaft, and short point: inner length 45 (43–46; *n* = 3); outer length 35 (32–38; *n* = 3); inner root 13 (12–14; *n* = 3); outer root 3 (3–4; *n* = 3); point 3 (3–4; *n* = 3). Ventral bar sickle-shaped, supporting membrane well defined: total length 23 (21–24; *n* = 4); median width 5 (3–6; *n* = 3); total width 11 (11–12; *n* = 3). Dorsal bar triangular, supporting membrane poorly sclerotised, with lateral rod-shaped formation: total length 22 (21–24; *n* = 4); median width 6 (6–7; *n* = 3); total width 9 (9–10; *n* = 3). Hooks 7 pairs, dissimilar in size; hook lengths (*n* = 3): pair I 16 (15–17); pair II 25 (22–27); pair III 23 (22 – 24); pair IV 32 (30–33); pair V 10 (8–12); pair VI 25 (24–27); pair VII 28 (27–28). Vagina weakly sclerotised tube, usually visible with spoon-shaped distal part; 11 (10–12; *n* = 2). MCO comprising basally articulated copulatory tube, accessory piece; total length 17 (16–18; *n* = 7). Copulatory tube arcuate, ladle-like base with a tear-like basal process; tube-trace length 20 (19–24; *n* = 7); base length 5 (4–5; *n* = 5); base width 3 (3–4; *n* = 5). Accessory piece well sclerotised, with small proximal and larger distal loop, projection reaching the half tangentially, distal part of the accessory piece a sheath guiding the copulatory tube.

*Molecular characterisation:* Fragments of 18S-ITS1 (776 bp), and 28S rDNA (760 bp) regions were successfully sequenced for two specimens of *A. caputfemoris* n. sp.. The 18S-ITS1 and 28S rDNA sequences obtained for this species did not show any intraspecific variation.

### Remarks

*Annulotrema caputfemoris* n. sp. and *A. arcum* n. sp. possess similar MCO; the differentiation from *A. arcum* n. sp. is given in the Remarks section of the species above. An important morphological feature is the visibility of a vagina in *A. caputfemoris* n. sp.

## *Annulotrema retortum* Kičinjaová, Přikrylová and Smit n. sp. (Fig. 3)

**Figure 3:**
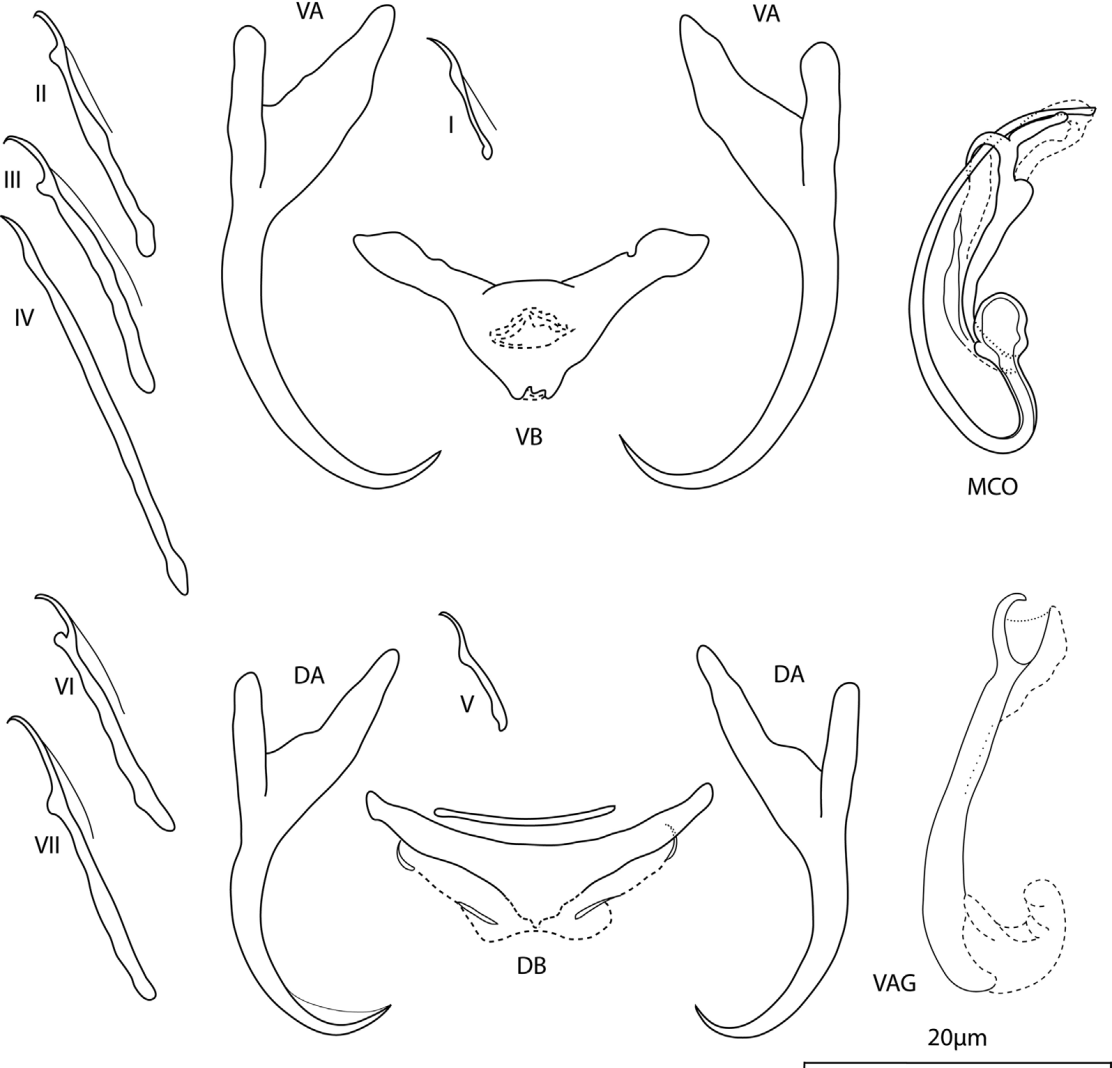
Line drawings of hard parts of *Annulotrema retortum* n. sp. ex *Micralestes acutidens* VA – ventral anchor, VB – ventral bar, DA – dorsal anchor, DB – dorsal bar, MCO – male copulatory, VAG – vagina.

urn:lsid:zoobank.org:act:632ED246-D67C-4C1E-8083-31A00758B29A

*Type host: Micralestes acutidens* (Peters, 1852)

*Type locality:* Phongolo River, South Africa (Bridge out - bridge outside of the Ndumo Game Reserve, 27°2.23482′S, 32°16′E)

*Site:* Gill lamellae

*Type specimens:* Holotype, five paratype specimens, and hologenophore (NMB P-1053, 1054, 1055, 1056), five paratype specimens, and one paragenophore (IPBCAS M-802).

*Representative DNA sequences*: GenBank: PQ279568 (18S-ITS1 rDNA) and PQ279574 (28S rDNA).

*Etymology:* The specific name “*retortum*” refers to the bent copulatory tube of the MCO. A retort is a laboratory device used for distillation, a blown glass flask with an extended bent neck.

*Infestation indices*: *P* = 30%, IF = 1–4, MI = 2, MA = 0.6

*Description*: Body length 283 (240–343; *n* = 5); greatest width 82 (69–92; *n* = 5). Ventral anchors with short inner root slightly recurved at its terminal part, well developed elongated outer root, elongated curved shaft, and short point: inner length 32 (29–34; *n* = 6); outer length 31 (29–32; *n* = 6); inner root 10 (9–11; *n* = 6); outer root 4 (3–6; *n* = 6); point 5 (4–6; *n* = 6). Dorsal anchors with short inner root having recurved terminal half, well developed elongated outer root, slightly curved shaft, and short point: inner length 26 (25–28; *n* = 6); outer length 24 (23–26; *n* = 6); inner root elongated 10 (8–11; *n* = 6); outer root 5 (4–5; *n* = 6); point 3 (3–4; *n* = 6). Ventral bar triangular, base with a triangular gap-like structure; total length 23 (20–25; *n* = 6); median width 6 (5–8; *n* = 4); total width 9 (9–10; *n* = 5). Dorsal bar triangular, supporting membrane well defined, with lateral rod-shaped formation; total length 23 (22–25; *n* = 4); median width 6 (5–6; *n* = 4); total width 9 (7–10; *n* = 4). Hooks 7 pairs, dissimilar in size; hook lengths (*n* = 8): pair I 11 (10–14); pair II 17 (15–19); pair III 17 (15–18); pair IV 25 (23–27); pair V 10 (9–11); pair VI 17 (16–19); pair VII 18 (17– 20). Vagina weakly sclerotised, elongated tube with pincer-like distal part and pouch-like proximal part (like a smurf hat); total length 13 (10–16; *n* = 8); tube-trace length 17 (14–25; *n* = 8). MCO comprising basally articulated copulatory tube, accessory piece; total length 25 (24–27; *n* = 6). Copulatory tube an arcuate slender tube, sharply bent (cca 45°) on its proximal half, with thick-walled ovate base; tube-trace length 45 (44–47; *n* = 6); base length 5 (4–5; *n* = 4); base width 3 (3–4; *n* = 4). Accessory piece biramous (one branch well sclerotised, one branch membranous), with short rounded process on its terminal half. Distal bifurcation of well sclerotised branch: one protuberance pointed, looping the copulatory tube, connecting to the membranous part, second one rounded, guiding the copulatory tube).

*Molecular characterisation:* Fragments of 18S-ITS1 (948 bp), and 28S rDNA (818 bp) regions were successfully sequenced for four specimens of *A. retortum* n. sp. The 18S-ITS1 and 28S rDNA sequences obtained for this species did not show any intraspecific variation.

### Remarks

*Annulotrema retortum* n. sp. closely resembles *A. edeense* (grammatical emendation of *A. edeensis* Birgi, 1988 [61]) from an unspecified host, *Micralestes* sp., and *A. sangmelinense* (grammatical emendation of *A. sangmelinensis* Birgi, 1988 [61]) from *Micralestes humilis* Boulenger, both found in Cameroon. Birgi [5] described these two species, *A. edeense* and *A. sangmelinense,* on only three specimens and did not deposit any type-material. *Annulotrema edeense* and *A. sangmelinense* depicted by Birgi in 1988 and *A. retortum* n. sp. show minimal differences in the morphology and measurements of anchor-bar complexes. Primarily the phase-contrast microscopy revealed a well sclerotised supporting membrane of the dorsal bar in *A. retortum* n. sp. *Annulotrema sangmelinense* differs from *A. retortum* n. sp. by possessing a bow-shaped MCO with an uniramous articulated accessory piece with a shuttle-like distal part. The vagina of *A. sangmelinense* was not recorded. Comparative morphology of reproductive organs of *A. edeense* and *A. retortum* n. sp. showed very small differences: the short rounded process of the accessory piece is lacking in *A. edeense*, and the bifurcation of the accessory piece is not apparent. Even though the similarity of both species, *A. edeense* and *A. retortum* n. sp., is evident, they cannot be officially synonymised as the type material of *A. edeense* is unavailable to study. We consider the comparison of specimens found on the type locality and type host to be the best way of verifying the status of *A. edeense*. Thus we designated *A. retortum* n. sp. as a new species.

## *Annulotrema strepsiceros* Kičinjaová, Přikrylová and Smit n. sp. (Fig. 4)

**Figure 4:**
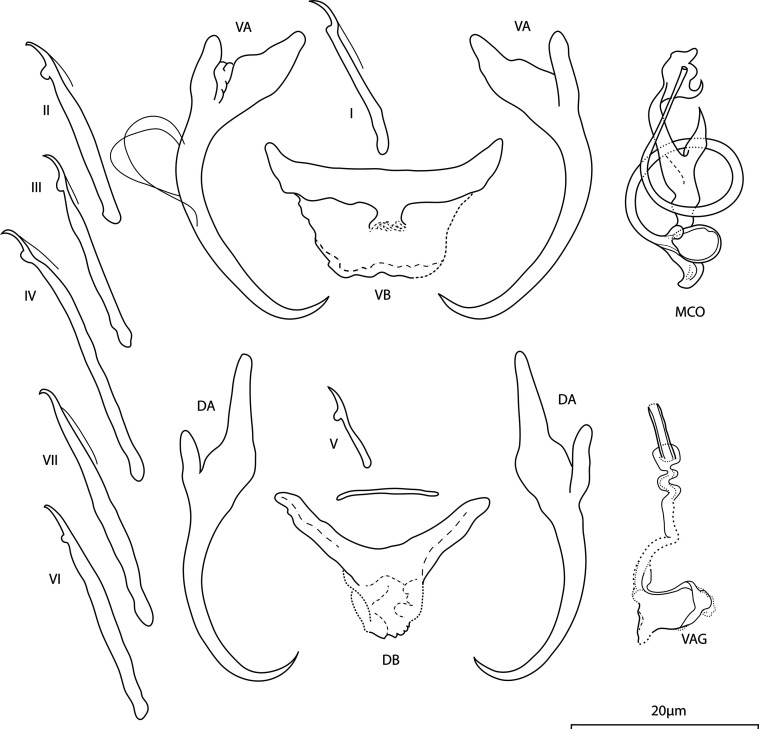
Line drawings of hard parts of *Annulotrema strepsiceros* n. sp. ex *Brycinus imberi.* VA – ventral anchor, VB – ventral bar, DA – dorsal anchor, DB – dorsal bar, MCO – male copulatory, VAG – vagina.

urn:lsid:zoobank.org:act:E95E0C85-5F2C-4AB5-96A6-B43478A223AF

*Type host: Brycinus imberi* (Peters, 1852)

*Type locality:* Phongolo River, South Africa: Broken bridge (Ndumo Game Reserve, outflow of Nyamithi Pan, 26°52.961833′S, 32°18.678′E)

*Site:* Gill lamellae

*Type specimens:* Holotype, one paratype, hologenophore and one paragenophore (NMB P-1057, 1058, 1059, 1060), five paratype specimens and one paragenophore (IPBCAS M-803).

*Representative DNA sequences*: GenBank: PQ279569 (18S-ITS1 rDNA) and PQ279575 (28S rDNA).

*Comparative material examined:* Syntypes (M.T. 35.918) of *A. alestesnursi* from *Alestes nurse* (= *Brycinus nurse*) (Uganda); Syntypes of *A. allogravis* (M.T. 35.716) from *Alestes imberi* (= *B. imberi*) (Tanzania)

*Etymology:* The specific name derived from Latin refers to the copulatory tube of the MCO resembling twisted horns of Greater Kudu (*Tragelaphus strepsiceros*), the popular antelope of South Africa.

*Infestation indices*: *P* = 80%, IF = 3–14, MI = 5.5, MA = 4.4

*Description*: Body length 309 (255–358; *n* = 6); greatest width 89 (76–121; *n* = 6). Ventral anchors with short inner root slightly recurved at its terminal part, well developed outer root, elongated bent shaft, and short point, anchor filaments visible: inner length 38 (36–40; *n* = 7); outer length 39 (38–44; *n* = 7); inner root 9 (7–11; *n* = 7); outer root 4 (3–6; *n* = 7); point 6 (4–6; *n* = 7). Dorsal anchors with elongated inner root having recurved terminal half, well developed outer root, slightly curved shaft, and short point: inner length 44 (42–48; *n* = 7); outer length 34 (33–38; *n* = 7); inner root 14 (12–15; *n* = 7); outer root 3 (2–3; *n* = 7); point 4 (4–5; *n* = 7). Ventral bar boat-shaped, supporting membrane well defined; total length 28 (26–29; *n* = 7); median width 6 (4–8; *n* = 6); total width 12 (11–14; *n* = 6). Dorsal bar saddle-shaped, supporting membrane poorly sclerotised, with lateral rod-shaped formation; total length 26 (24–29; *n* = 7); median width 12 (10–15; *n* = 6); total width 19 (16–22; *n* = 6). Hooks 7 pairs, dissimilar in size; hook lengths (*n* = 5): pair I 20 (18–21); pair II 25 (24–27); pair III 29 (27–31); pair IV 35 (34–36); pair V 12 (11–13); pair VI 25 (24 – 27); pair VII 29 (28–30). Vagina weakly sclerotised, sac-shaped vaginal aperture, proximal part visible or non-visible; 18 (8–25; *n* = 7). MCO comprising copulatory tube, accessory piece; total length 29 (27–30; *n* = 7). Copulatory tube an elongate solid coil of one medially looped ring, spring-like, coiled around the median part of the accessory piece, base of the copulatory tube ovate, with petal-shaped basal process; tube-trace length 64 (63–65; *n* = 7); base length 6 (6–7; *n* = 7); base width 4 (4–5; *n* = 7). Accessory piece basally articulated, massive, noticeable thorn-like structure arising medially, an extension with two terminal branches (one claw-shaped and one dull) supporting the distal part of the copulatory tube.

*Molecular characterisation:* Fragments of 18S-ITS1 (753 bp), and 28S rDNA (775 bp) regions were successfully sequenced for three specimens of *A. strepsiceros* n. sp. The 18S-ITS1 and 28S rDNA sequences obtained for this species did not show any intraspecific variation.

### Remarks

The morphology of the haptoral sclerites and the MCO *Annulotrema strepsiceros* n. sp. closely resembles the “small specimen” of *A. alestesnursi* Paperna, 1973 from *B. nurse* (Uganda) as reported by Paperna in 1979 (Plate XXXI: 7 and 8) [38] and *A. allogravis* Paperna, 1973 from *B. imberi* (Tanzania) [37]*.* The MCO of all species mentioned above is a coil of one ring with a basally articulated accessory piece with a medial and terminal spike. In the morphological evaluation of syntypes of *A. alestesnursi* (M.T. 35.918) and syntypes of *A. allogravis* (M.T. 35.716), clear differences in the shape of the distal part of the accessory piece were observed, which is flying bird-shaped in *A. alestesnursi* and possessing one sharp terminal spike in *A. allogravis* (*vs.* more obtuse process and lateral spike in *A. strepsiceros* n. sp.). Moreover, the visibility of a vagina in *A. strepsiceros* n. sp. clearly distinguishes the new species from the above species.

The comparison of the measurements of *Annulotrema* spp. found in the present study is presented in Table S1, and the raw data of measurements are available in Table S2.

### Molecular characterisation and phylogenetic reconstruction

**Figure 5:**
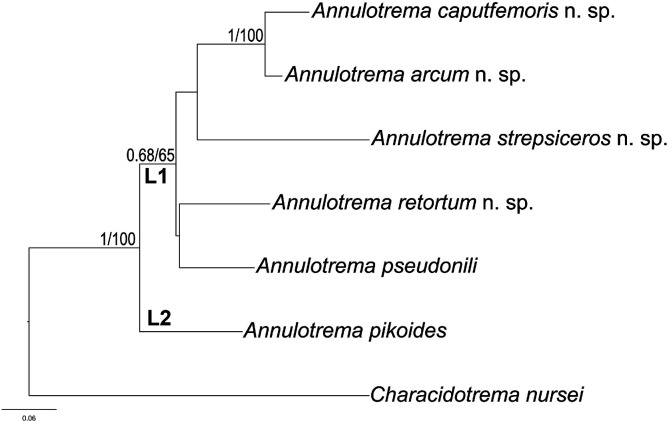
Maximum likelihood (ML) tree inferred from 28S rDNA sequence dataset (729 bp) depicting the phylogenetic relationships of *Annulotrema* species analysed in the present study. The numbers along branches indicate Bayesian posterior probabilities and ML bootstrap percentages. Bootstraps with low support (below 50) are not shown.

The results of a BLASTn search (November 2023) of the 18S-ITS1 and 28S rDNA fragments revealed no identical entries in GenBank. Uncorrected *p*-distances between *Annulotrema* spp. analysed in the present study ranged from 12.8% to 19.6% for 18S-ITS1 and from 5.6% to 18.6% for 28S rDNA (Table 3). The topologies of the ML and BI trees were congruent; therefore, the consensus tree based on the ML topology, with support values from both algorithms, is shown (Fig. 5

). *Annulotrema* spp. were found to form two clades, clade 1 consists of five *Annulotrema* spp*.*, two sister taxa, and the closest related taxa of all included in the analysis, *A. caputfemoris* n. sp. and *A. arcum* n. sp., *A. strepsiceros* n. sp., and a not well supported cluster of *A. retortum* n. sp and *A. pseudonili* (Fig. 5). The second clade is represented by a single species, *A. pikoides*.

**Table 3:** Matrix of pairwise genetic distances (*p*-distance) of *Annulotrema* spp. investigated in this study based on the alignment of 18S-ITS1 (above diagonal) and 28S rDNA (below diagonal) nucleotide sequences.

		1	2	3	4	5	6
1	*Annulotrema arcum* n. sp.		*—*	*—*	*—*	*—*	*—*
2	*Annulotrema caputfemoris* n. sp.	5.6		17.6	15.8	18.4	12.8
3	*Annulotrema pikoides*	17.1	18.4		18.5	19.6	16.8
4	*Annulotrema pseudonili*	13.3	15.4	15.7		15.5	16.1
5	*Annulotrema retortum* n. sp.	14.4	15.4	16.1	12.8		18.5
6	*Annulotrema strepsiceros* n. sp.	16.9	18.4	18.6	18.1	17.0	

## Discussion

Identification of the African dactylogyrid monopisthocotyleans is traditionally based on the morphometry of hard parts of the haptor and reproductive system [49]. In one of the earlier studies, Kičinjaová *et al.* [25] pointed out the existence of morphotypes based on the morphology of the sclerotised structures of some *Annulotrema* spp., mainly of the male copulatory organ. These authors also promoted the notion that the similarities of the MCOs can be regarded as a feature indicating a close relationship among these species. Similarly, the MCO concept can be applied to species of *Dactylogyrus* Diesing, 1850 [50, 51], while on the other hand, the haptoral hard parts concept is used for *Quadriacanthus* Paperna, 1961 [12]. In the current study, *A. arcum* n. sp. and *A.* *caputfemoris* n. sp. share morphologically very similar types of MCO and the phylogenetic analysis confirmed their apparent close relationship by clustering as sister taxa within clade 1, and by being the closest related species based on the lowest observed *p*-distances of 28S rDNA sequences. Although the nodal support of *A. strepsiceros* n. sp. clustering together with *A. arcum* n. sp. and *A.* *caputfemoris* n. sp. was low, there are morphological similarities between these species supporting the molecular results. The MCOs of all three species above share the same morphotype by having an articulated accessory piece guiding the distal part of the copulatory tube. Moreover, all three species are described from the same host, *B. imberi*, providing further ecological support as well. The findings of the present study using an integrated approach (morphological and molecular analysis) confirm the morphology of the MCO as the most important feature to distinguish the representatives of *Annulotrema* at the species level. This conclusion fully aligns with the earlier studies that promoted the MCO-type concept [2, 3, 4]. Additionally, other studies suggest that species with a certain type of MCO often share a certain host [12, 46] and that leads to a necessity of diversification of parasites sharing the same host to prevent interspecific hybridisation. Nevertheless, only a more detailed phylogenetic study based on combined molecular data of the 18S-ITS1 and 28S rDNA regions and newly produced sequences for more *Annulotrema* spp. can shed light on the understanding of the phylogenetic relationship within the genus and between the other African dactylogyrid genera.

Řehulková *et al.* [49] pointed out that the examination of a tiny proportion of known fish species results in the currently low numbers of monopisthocotylean parasites described in Africa, with African tetras being one of the examples. To date, only 16 of 120 spp. of African tetras have been examined and confirmed as hosts of monopisthocotylean parasites representing six genera (e.g. *Alestes, Brycinus, Hemigrammopetersius, Hydrocynus, Micralestes*, and *Phenacogrammus*). Species of *Brycinus* and *Hydrocynus* represent the hosts with the highest species richness of dactylogyrid parasites reported. Primacy with 14 spp. belongs to *Hydrocynus forskahlii* (Cuvier) [7]. Another species of *Hydrocynus*, *H. vittatus* is known to be parasitised by eight species and two subspecies, with the present study reporting two of the species, *A. pikoides* and *A. pseudonili.* The study on parasites of *H. vittatus* by Mabika *et al.* [28] from Lake Kariba (Zimbabwe) reported on two species of *Annulotrema*, but only identified them as sp. 1 and 2. The following work (from the same host and locality), of Kičinjaová *et al.* [24] reports on three *Annulotrema* spp., including two new species descriptions. The later work by Mabika *et al.* [28] also reported on and confirms three species from *H. vittatus*, with two of them (*A. pseudonili* and *A. bracteatum*) overlapping with Kičinjaová *et al.* [24].

Species of *Brycinus* are known to host monopisthocotylean of *Annulotrema* and *Characidotrema*, with the latter being exclusive to species of Alestidae, while *Annulotrema* spp. infect hosts of a further two families, the Distichodontidae and Hepsetidae [24, 24, 25, 33, 48]. Until now, *B. nurse* was the species with the highest number of monopisthocotylean species (four *Annulotrema* spp. and four *Characidotrema* spp.). The present study describes three new species from *B. imberi*, which is now host to eight monopisthocotylean species, five *Annulotrema* spp. and three *Characidotrema* spp., respectively. The findings of the present study indicate that the species of *Brycinus* would be an interesting target to be studied for its parasite fauna. To date, only seven out of 36 species of the commercially important pan-African genus *Brycinus* are known to be hosts of dactylogyrid parasites, demonstrating that the parasite diversity of these hosts is still poorly understood.

Currently, *Annulotrema* includes 50 nominal species and two subspecies recorded on representatives of the Alestidae, Distichodontidae, and Hepsetidae [23, 24, 25, 33, 48]. The work of Birgi in 1988 [5] presented descriptions of 15 *Annulotrema* spp. from Cameroon. This work makes up a significant part of all discoveries of the currently known *Annulotrema* spp. (almost 1/3). Unfortunately, the publication does not mention the deposition of type materials. Also, our efforts to find the type material of these *Annulotrema* spp. were unsuccessful, which supports our assumption of the non-existence of any type material. Such a situation might lead to the decision to mark all dactylogyrid species described in Birgi’s publication from 1988 as *nomina dubia.* Only a study of the type hosts from the type locality would provide answers and verify the validity of all these *Annulotrema* spp. Therefore, we propose that a complete revision of *Annulotrema* spp. is necessary due to low quality/incomplete original description together with the absence of type material. In addition, some morphometric features (morphology of MCO, absence of supporting membranes of the ventral and dorsal bars) together with host preferences need to be revisited to confirm the affiliation of certain species of *Annulotrema*, especially those found on species of the Distichodontidae and Hepsetidae.

## Conclusions

The current study brings the description of four new *Annulotrema* spp. and provides the first molecular data for six *Annulotrema* spp. Despite limited data available for the phylogenetic comparison, the analysis confirmed the existence of lineages within the *Annulotrema*. However, only once sequencing of more species of the genus is available for further phylogenetic analysis will we be able to fully appreciate the relationships among congeners and between other genera of African dactylogyrids.

## Supplementary material

The supplementary material of this article is available at https://www.parasite-journal.org/10.1051/parasite/2024066/olm. ***Table S1*.** Comparison of the measurements of *Annulotrema* spp. found in the present study, mean and the range in brackets; the values are presented in µm. ***Table S2*.** Raw data of the measurements.
