# Supplementary material for: Species of Annulotrema (Monopisthocotylea, Dactylogyridae) parasitising African tetras (Characiformes, Alestidae) in the Phongolo River, South Africa with the description of four new species
Source: Parasite. 2024 Oct 31;31:67. doi: 10.1051/parasite/2024066 (PMC11527425; doi:10.1051/parasite/2024066)
Supplement: Supplementary file 1 — Table S1. Comparison of the measurements of Annulotrema spp. found in the present study, mean and the range in brackets; the values are presented in µm. [file parasite-31-67-s1.pdf]

Table S1 Comparison of the measurements of *Annulotrema* spp. found in the present study, mean and the range in the bracket, the values are presented in µm.

**Species**<br>**Measurements**	***A. arcum* n. sp**	***A. caputfemoris* n. sp.**	***A. retortum* n. sp.**	***A. strepsiceros* n. sp.**	***A. pikoides***	***A. pseudonili***
Body length	315<br>(285 – 342)	331<br>(326 – 340)	283<br>(240 – 343)	309<br>(255 – 358)	658<br>(566 – 746)	593<br>(480 – 706)
Body greatest width	131<br>(95 – 182)	123<br>(75 – 165)	82<br>(69 – 92)	89<br>(76 – 121)	107<br>(93 – 120)	167<br>(135 – 187)
VA inner length	49<br>(44 – 52)	41<br>(40 – 44)	32<br>(29 – 34)	38<br>(36 – 40)	45<br>(44 – 46)	40<br>(39 – 42)
VA outer length	48<br>(42 – 51)	40<br>(39 – 42)	31<br>(29 – 32)	39<br>(38 – 44)	41<br>(40 – 43)	46<br>(43 – 50)
VA inner root	12<br>(11 – 13)	12<br>(10 – 12)	10<br>(9 – 11)	9<br>(7 – 11)	12<br>(9 – 13)	12<br>(11 – 13)
VA outer root	5<br>(4 – 6)	4<br>(3 – 4)	4<br>(3 – 6)	4<br>(3 – 6)	5<br>(4 – 7)	6<br>(4 – 8)
VA point	5<br>(5 – 6)	4<br>(4 – 5)	5<br>(4 – 6)	6<br>(4 – 6)	7<br>(6 – 8)	9<br>(8 – 10)
DA inner length	54<br>(45 – 60)	45<br>(43 – 46)	26<br>(25 – 28)	44<br>(42 – 48)	46<br>(44 – 50)	50<br>(48 – 53)
DA outer length	41<br>(36 – 45)	35<br>(32 – 38)	24<br>(23 – 26)	34<br>(33 – 38)	39<br>(37 – 43)	40<br>(37 – 42)
DA inner root	17<br>(14 – 19)	13<br>(12 – 14)	10<br>(8 – 11)	14<br>(12 – 15)	15<br>(14 – 15)	21<br>(19 – 23)
DA outer root	4<br>(3 – 5)	3<br>(3 – 4)	5<br>(4 – 5)	3<br>(2 – 3)	6<br>(5 – 6)	6<br>(5 – 8)

DA point	4<br>(3 – 5)	3<br>(3 – 4)	3<br>(3 – 4)	4<br>(4 – 5)	6<br>(6 – 8)	8<br>(7 – 10)
VB total length	26<br>(23 – 28)	23<br>(21 – 24)	23<br>(22 – 25)	28<br>(26 – 29)	31<br>(30 – 32)	32<br>(27 – 34)
VB median width	8<br>(4 – 11)	5<br>(3 – 6)	6<br>(5 – 8)	6<br>(4 – 8)	6<br>(4 – 8)	6<br>(4 – 9)
VB total width	13<br>(13 – 14)	11<br>(11 – 12)	9<br>(9 – 10)	12<br>(11 – 14)	13<br>(11 – 17)	20<br>(18 – 23)
DB total length	26<br>(24 – 27)	22<br>(21 – 24)	23<br>(22 – 25)	26<br>(24 – 29)	28<br>(27 – 29)	32<br>(29 – 36)
DB median width	6<br>(5 – 9)	6<br>(6 – 7)	6<br>(5 – 6)	12<br>(10 – 15)	8<br>(8 – 9)	14<br>(14 – 14)
DB total width	11<br>(10 – 13)	9<br>(9 – 10)	9<br>(7 – 10)	19<br>(16 – 22)	12<br>(11 – 13)	21<br>(18 – 23)
MH pair I	20<br>(18 – 21)	16<br>(15 – 17)	11<br>(10 – 14))	20<br>(18 – 21)	16<br>(13 – 18)	18<br>(17 – 20)
MH pair II	30<br>(29 – 31)	25<br>(22 – 27)	17<br>(15 – 19)	25<br>(24 – 27)	22<br>(21 – 22)	23<br>(19 – 25)
MH pair III	29<br>(27 – 30)	23<br>(22 – 24)	17<br>(15 – 18)	29<br>(27 – 31)	27<br>(25 – 30)	27<br>(25 – 28)
MH pair IV	36<br>(33 – 39)	32<br>(30 – 33)	25<br>(23 – 27)	35<br>(34 – 36)	33<br>(32 – 33)	32<br>(30 – 34)
MH pair V	14<br>(12 – 16)	10<br>(8 – 12)	10<br>(9 – 11)	12<br>(11 – 13)	12<br>(9 – 14)	15<br>(13 – 16)
MH pair VI	28<br>(26 – 30)	25<br>(24 – 27)	17<br>(16 – 19)	25<br>(24 – 27)	25<br>(22 – 27)	27<br>(26 – 28)
MH pair VII	30<br>(29 – 33)	28<br>(27 – 28)	18<br>(17 – 20)	29<br>(24 – 27)	22<br>(21 – 24)	33<br>(31 – 35)

VAG total length	not observed	11 (10 – 12)	13 (10 – 16)	18 (8 – 25)	not observed	not observed
VAG tube-trace length			17 (14 – 25)			
MCO total length	20 (19 – 21)	17 (16 – 18)	25 (24 – 27)	29 (27 – 30)	57 (53 – 64)	31 (24 – 39)
MCO tube-trace length	30 (29 – 30)	20 (19 – 24)	45 (44 – 47)	64 (63 – 65)	72 (68 – 75)	82 (73 – 92)
MCO base length	6 (5 – 7)	5 (4 – 5)	5 (4 – 5)	6 (6 – 7)	7 (7 – 8)	12 (11 – 13)
MCO base width	4 (4 – 4)	3 (3 – 4)	3 (3 – 4)	4 (4 – 5)	4 (3 – 5)	5 (5 – 5)

Abbreviations: VA ventral anchor, DA dorsal anchor, VB ventral bar, DB dorsal bar, MH marginal hook, VAG vagina, MCO male copulatory organ
